# Gametophytic Selection in *Arabidopsis thaliana* Supports the Selective Model of Intron Length Reduction

**DOI:** 10.1371/journal.pgen.0010013

**Published:** 2005-08-05

**Authors:** Cathal Seoighe, Chris Gehring, Laurence D Hurst

**Affiliations:** 1 Institute of Infectious Disease and Molecular Medicine, University of Cape Town, Rondebosch, South Africa; 2 Department of Biotechnology, University of the Western Cape, Bellville, South Africa; 3 Department of Biology and Biochemistry, University of Bath, Somerset, United Kingdom; North Carolina State University, United States of America

## Abstract

Why do highly expressed genes have small introns? This is an important issue, not least because it provides a testing ground to compare selectionist and neutralist models of genome evolution. Some argue that small introns are selectively favoured to reduce the costs of transcription. Alternatively, large introns might permit complex regulation, not needed for highly expressed genes. This “genome design” hypothesis evokes a regionalized model of control of expression and hence can explain why intron size covaries with intergene distance, a feature also consistent with the hypothesis that highly expressed genes cluster in genomic regions with high deletion rates. As some genes are expressed in the haploid stage and hence subject to especially strong purifying selection, the evolution of genes in *Arabidopsis* provides a novel testing ground to discriminate between these possibilities. Importantly, controlling for expression level, genes that are expressed in pollen have shorter introns than genes that are expressed in the sporophyte. That genes flanking pollen-expressed genes have average-sized introns and intergene distances argues against regional mutational biases and genomic design. These observations thus support the view that selection for efficiency contributes to the reduction in intron length and provide the first report of a molecular signature of strong gametophytic selection.

## Synopsis

Genes are odd things. Small proteins are often encoded by big genes. In the process, much of the excess material has to be cut out and thrown away. The size of the parts that are discarded (introns) differs greatly between genes. Why should this be so? The authors test three different ideas, making use of the unusual fact that in plants genes are expressed in pollen. As pollen has only one copy of every gene, natural selection is expected to work somewhat better. The authors find that the non-coding parts of genes that are especially active in pollen are particularly small. They also find that being active in pollen tends to make introns small. This provides strong support for the idea that small introns are the result of selection to reduce costs of making too much material that is only going to be thrown away.

## Introduction

Selection for efficiency has been proposed to explain the reduced intron lengths of broadly or highly expressed genes in several animal systems [1–5]. Because of the energetic cost of transcription [4–6], which is proportional to the length of the transcript and the amount of the transcript that is produced, highly expressed genes are likely to experience greater selective pressure for a reduction in transcript length. This model sees long introns in weakly expressed genes as the result of weakened negative selection. This interpretation of the negative correlation between intron size and gene expression level [1–5] has recently been challenged. The genomic design hypothesis suggests that the shorter introns of highly expressed genes may not be the result of purifying selection, but instead reflect a reduced level of epigenetic regulation in housekeeping genes, which are often expressed at high levels [7]. Under this hypothesis, selection actively favours the accumulation of longer introns in less highly expressed genes because many of these genes are tissue specific and require greater levels of epigenetic regulation. This is supported by the fact that intergenic distances also tend to be reduced in the vicinity of highly expressed genes [2,7,8], an observation that is not explained by the transcriptional efficiency model. Moreover, if one controls for intergene distance, it is as yet unclear whether, in humans, highly expressed genes have small introns as reports are contradictory [2,7]. Hence, the relevance of the transcriptional efficiency model is currently uncertain. The correlation between intergene distance and intronic size has also been interpreted as evidence for a regional mutational bias, coupled with neutral evolution [2]. Indeed, regions of high compaction tend to be GC rich [2,8] and hence regions of high recombination rates [9]. If recombination induces deletions, then a simple mutational bias/neutral model can be considered.

Owing to the fact that it has abundant genes that are haploid expressed, *Arabidopsis thaliana* provides a novel testing ground to examine these conflicting viewpoints. Strong selection at the gametophytic stage, owing to haploid exposure of recessive mutations and/or to strong pollen competition [10–12], has been proposed as a key aspect of plant evolutionary biology resulting in the purging of deleterious mutations in genes that are transcribed in the growing pollen tube [13,14]. A transcriptional cost view of intron length variation predicts that this strong purifying selection should cause a reduction in intron lengths in genes that are expressed in pollen compared to genes that are expressed elsewhere and that this reduction should be most pronounced in the most highly expressed genes.

## Results/Discussion

**Figure 1:**
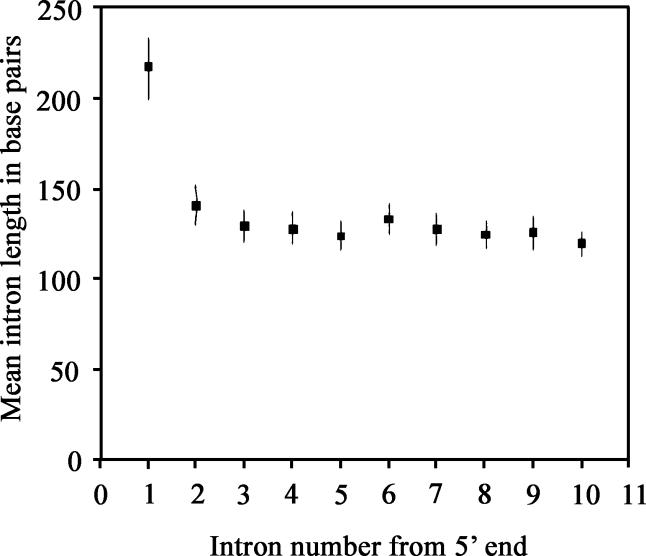
Mean Intron Length as a Function of Intron Position, Counting from the 5′ End of the Gene Intron length was nearly constant for introns 5 to 10. Proximity to the 3′ end of the gene was not correlated with intron length. Error bars show ± twice the standard error. The data shown are for genes with exactly ten introns so that positional effects from the 3′ ends can also be assessed.

Introns, particularly those toward the 5′ ends of genes, may often have regulatory functions [15]. The lengths of introns in animals have been shown to decrease as a function of the position of the intron, counting from the 5′ end, and to depend to some extent on the breadth of expression (i.e., the number of tissues in which the gene is expressed [5]). We find a similar reduction in intron length as a function of intron position in *Arabidopsis* (Figure 1). In order to reduce the impact of positional effects and regulatory elements associated with proximal introns, we restricted our analysis of intron lengths of genes that are expressed in pollen to distal introns (from intron 5 to intron 10) since they are less likely to have a role in regulation (our analyses were not sensitive to the cut-off used to classify distal introns).

**Figure 2:**
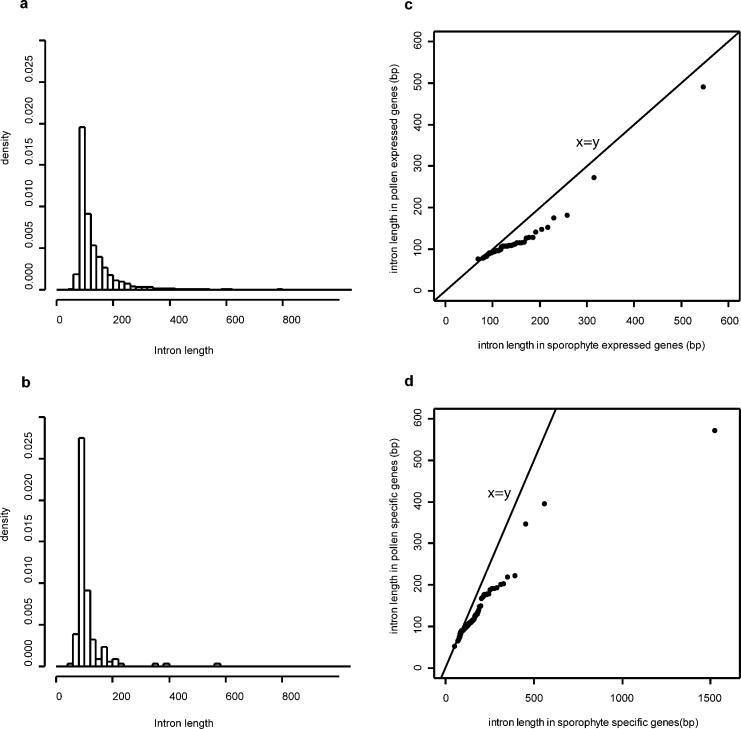
Histograms and Quantile–Quantile Plots of Mean Distal Intron Length (A) Histogram for genes expressed in the sporophyte microarray datasets. (B) Histogram for genes expressed in the gametophyte but not the sporophyte microarray datasets. (C) Quantile–quantile plot of introns from all pollen-expressed and all sporophyte-expressed genes derived from the SAGE dataset. Quantiles of the intron length distributions for genes expressed in the gametophyte and sporophyte are on the *x*- and *y*-axes, respectively. (D) Quantile–quantile plot of introns from pollen-specific and sporophyte-specific genes derived from the microarray dataset.

Using publicly available serial analysis of gene expression (SAGE) data, we compared intron lengths between genes that are expressed in pollen and the sporophyte. A summary of the dataset is provided in Table 1. The average intron length for the pollen genes was 107.7 base pairs (bp), compared to 123.4 bp for introns from genes expressed in at least one of four sporophyte conditions (*p* = 0.0002)*.* In spite of significant differences in means, the mode of the distribution of intron lengths remained approximately the same in both groups and for all intron positions. Comparison of the distributions of intron lengths shows that there were fewer longer introns among the genes that were expressed in the gametophyte compared to the sporophyte, as indicated by curvature away from the diagonal in a quantile–quantile plot (Figure 2). We also compared intron lengths between genes expressed in the sporophyte and gametophyte with expression level as a covariate, using expression levels from the pollen SAGE dataset and the largest [16] of the four sporophyte SAGE datasets in the study. We found significant evidence for both a negative correlation between intron length and gene expression level (*p* = 0.01) and a reduced intron length in genes expressed at a given level in the gametophyte compared to the sporophyte (*p* = 0.001). This latter result suggests that introns from genes expressed in pollen remain shorter than introns from genes in the sporophyte when we control for expression level.

**Table 1:**
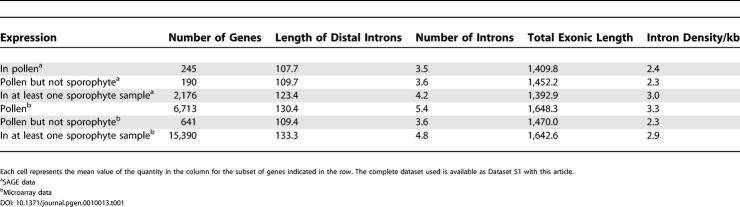
Summary of the Dataset

Each cell represents the mean value of the quantity in the column for the subset of genes indicated in the row. The complete dataset used is available as Dataset S1 with this article.

^a^SAGE data

^b^Microarray data

Might the reduced intron lengths of genes expressed in pollen be sensitive to the method of measurement of gene expression? We compared intron lengths between genes that are expressed in pollen but not in the sporophyte and vice versa using microarray data from the Expression Atlas of *Arabidopsis* Development [17]. The mean intron lengths for pollen-specific genes was 109.4 bp compared to 134.7 bp for the genes expressed in the sporophyte but not in pollen (*p* = 3 × 10^−9^). The expression level of the pollen-specific genes was higher, on average, than for genes that were expressed in pollen and the sporophyte. If expression level in pollen is included as a covariate, the length of introns remained significantly lower in genes that are specific to pollen compared to genes that are specific to sporophyte (*p* = 5 × 10^−5^). Introns from genes that were highly expressed in pollen were also significantly shorter than introns from genes that were highly expressed in at least one sporophyte sample, regardless of whether the gene was specific to pollen or expressed in both pollen and sporophyte (*p* = 0.0007).

The reduction in intron lengths in genes expressed in the pollen SAGE dataset did not appear to be affected by whether the genes were also expressed in the sporophyte, illustrating the potential impact of strong gametophytic selection on sporophyte evolution. In the SAGE dataset, genes that were specific to pollen and genes that were expressed in pollen as well as one of the sporophyte datasets had similar average intron lengths (99.1 bp and 109.7 bp; *n* = 13 and *n* = 58, respectively; *p* = 0.93), while in both cases the introns were significantly or marginally significantly shorter than introns of genes expressed in the sporophyte but not expressed in pollen (*p* = 0.06 and *p* = 0.0009). This additionally provides evidence that the observed difference in intron lengths between genes expressed in pollen and the sporophyte is not the result of a lack of intronic regulatory elements in genes that are expressed exclusively in pollen. Contrary to the results from SAGE, the reduction in intron lengths was confined to genes that were specific to pollen in the microarray datasets, possibly due to hybridisation cross-reactivity between homologous genes. Pollen has a high proportion of genes that appear to be expressed in pollen only [18]. The reduced intron lengths observed in such genes is not in keeping with the genomic design argument that suggests that regulation of narrowly expressed genes is responsible for their longer introns compared to broadly and highly expressed genes.

To test whether altered rates of insertion or deletion or a higher gene density in the genomic regions containing the genes that are expressed in pollen could be responsible for the reduced intron lengths, we calculated the average intron lengths of the closest genomic neighbours of the pollen genes from the SAGE dataset. The mean intron length of the neighbouring genes was not significantly different to the mean for all genes (132.5 bp compared to 134.8 bp, *p* = 0.14). The mean intron length for genes expressed in pollen remained significantly below the mean for genes expressed in the sporophyte, considering only the closest sporophyte-expressed neighbour for each gene expressed in pollen (*p* = 0.01). Thus, regional genomic effects evoked by the genomic design hypothesis [7] and the mutational bias hypothesis are not likely to be the cause of the reduced intron lengths of genes expressed in pollen. Furthermore, although the mean length of flanking regions was slightly greater for genes that were expressed in the sporophyte compared to pollen, the difference was not statistically significant (1,946 bp and 1,762 bp, for sporophyte and pollen, respectively; *p* = 0.57). Restricting to genes with at least five introns (the genes that contributed to this study), this difference is reduced, and the pollen genes, in fact, have slightly longer intergenic regions, although, again, the difference is not statistically significant (1,826 bp and 1,913 bp for sporophyte and pollen, respectively; *p* = 0.15).

The introns of genes that were highly expressed in at least one of the sporophyte expression sites in the study were significantly reduced in length compared to all genes expressed in the sporophyte (111.1 bp compared to 123.4 bp, *p* = 0.004). Under the genomic design hypothesis, this might be explained by the fact that highly expressed genes are often ubiquitous and do not require much regulatory information in introns or flanking regions [7]. A regional mutational bias model could explain the reduced introns if highly expressed genes are associated with high rates of deletions. Both of these hypotheses are supported by a positive correlation between the lengths of introns and flanking intergenic regions in human [2,7,8]. In contrast, for most genes there is very little correlation between intron length and the mean length of 5′ and 3′ flanking regions in *Arabidopsis* (Spearman ρ = 0.02, *p* = 0.09). Furthermore, the length of intergenic regions was not significantly correlated with mean expression in the sporophyte, and the intergenic regions flanking genes that were highly expressed in the sporophyte were not reduced in length (2,037.8 bp, compared to the mean of 1,986.6, *p* = 0.10). Thus, we find no evidence of a contribution from gene regulation or regional mutational effects to intron length variation in *Arabidopsis,* whereas the reduced intron lengths of genes expressed in pollen strongly support the transcriptional efficiency model.

Genes that were expressed in pollen had significantly lower intron densities (number of introns per kilobase of exon) than genes that were expressed in at least one of the sporophyte conditions (2.4 introns per kb compared to 3.0 introns per kb; *p* = 0.001). The genes that were the most highly expressed in pollen had an average intron density that was lower still (1.8 introns per kb), significantly lower than for the genes that were highly expressed in at least one of the sporophyte conditions (2.6 introns per kb; *p* = 0.01). It is possible that the reduced intron densities result from a disproportionate number of partially processed retroposed genes in the pollen gene dataset rather than from selection for efficiency. Although we cannot rule this out, if retroposition is indeed responsible we might expect an increased density of introns toward the 3′ end [19]. However, we find that the relative density of introns in the 3′ and 5′ halves of genes expressed in pollen is no different to genes expressed in the sporophyte (data not shown).

The debate over whether selection to reduce the cost of transcription is indeed responsible for the shorter intron lengths observed in highly and broadly expressed animal genes has remained unresolved [2,7]. Very high levels of competition at the gametophytic stage of plants provide a useful system in which selection hypotheses can be explored. Although natural selection acting on genes that are expressed at the haploid stage is thought to be an important aspect of plant evolutionary biology [13,14], the reduction in intron lengths that we observe in the genes that are expressed in pollen represents the first well-demonstrated example of the impact of gametophytic selection on the genome of a plant. At least in the case of the genes that are expressed in pollen, there is strong evidence that selection for efficiency, rather than genomic design or regional mutational bias, plays a major role in shaping intron content.

Patterns of genome evolution can differ significantly between outcrossing organisms and self-fertilizing organisms, such as *A. thaliana* [20]. Because *Arabidopsis* has most probably become highly self-fertilizing in the relatively recent past [21] and because insertions and deletions occur on a much longer time-scale than base substitutions [22,23], we expect the evolution of intron lengths in *Arabidopsis* to be dominated by outcrossing reproduction. However, even though heterozygosity is greatly reduced in self-fertilizing organisms so that most gametophytes carry identical alleles, gametophytic competition between transient heterozygotes resulting from de novo mutations still occurs and may well be sufficient to cause the observed reduction of intron lengths in genes expressed in pollen.

Is it conceivable that a slightly different model might apply, one in which speed rather than cost of transcription was important, owing to the fact that only one copy of the genome is present in pollen? If the increased time required to transcribe long introns rather than the energetic cost of transcription [3,6,24] is the primary selective force acting to reduce intron lengths, then it could be argued that the reduced availability of template in the gametophyte, rather than gametophytic selection, could explain intron length reduction in pollen genes. However, because several polymerases can be attached to the same template simultaneously [25,26], gene length need not have much, if any, impact on the steady-state capacity of the template to produce messenger RNA. The additional time required to transcribe longer genes may increase the activation time of a gene but is not expected to have a disproportionately large impact on highly expressed genes. In contrast, the energetic cost of transcription is a linear function of the amount of the transcript that is produced, irrespective of whether transcription is from one or two templates. Because the energetic cost of transcribing longer genes is the same in the gametophyte and sporophyte, we consider that the increased sensitivity to slight differences in fitness caused by strong gametophytic selection is responsible for the reduced length of the introns from genes that are expressed in pollen.

## Materials and Methods

Models of *A. thaliana* genes were extracted from version 5 of the annotated *Arabidopsis* genome downloaded from TIGR (ftp://ftp.tigr.org/pub/data/a_thaliana/ath1/PSEUDOCHROMOSOMES/). Genes for which more than one gene model was available (corresponding to alternative transcript isoforms) were omitted. SAGE gene expression data derived from pollen [27], seedlings [28], seedling roots [29], root [30], and seedling aerial tissue [16] were downloaded from the Gene Expression Omnibus (http://www.ncbi.nlm.nih.gov/geo) or from the source data of the original manuscripts. Only tags that were mapped to a single gene and genes to which only a single tag had been mapped were retained for each dataset. In each of the SAGE datasets, the 20% of genes with the highest tag counts were defined as highly expressed. We used only expression data from tags with counts of at least five for each dataset in order to ensure robust results and that the data were comparable between all of the datasets. All statistical tests were carried out in the R statistical computing environment (http://www.R-project.org).Two-tailed Wilcoxon Rank Sum tests were performed for all of the comparisons between sample means. We used robust regression to fit a linear model to intron lengths as a function of expression level and site of expression (sporophyte or gametophyte) considering all genes expressed in pollen and genes from the SAGE dataset representing the largest number of genes (constructed from the aerial part of the plant [16]). For the linear model, only genes from the sporophyte dataset that were not also present in the gametophyte dataset were considered. Gene expression levels in pollen and a range of sporophyte conditions (root, leaf, stem, hypocotyl, and seedling), estimated using the Affymetrix (Santa Clara, California, United States) ATH1 Arabidopsis Genome Array Gene Chip as part of the Expression Atlas of *Arabidopsis* Development [17], were obtained prior to publication with the kind permission of the authors. The data are available from the NASCArrays database (http://affymetrix.arabidopsis.info/narrays/experimentbrowse.pl; slide Ids ATGE_73_A/B/C, ATGE_3_A/B/C, ATGE_91_A/B/C, ATGE_28_A2/B2/C2, ATGE_2_A/B/C, ATGE_96_A/B/C). We used the mean value of the signal for each gene that was called present in the original analysis. For each condition, the top 20% of most highly expressed genes were defined as highly expressed.

## Supporting Information

Dataset S1 Gene Expression and Intron Length Data (2.2 MB TXT)

## Abbreviations

bp: base pair
SAGE: serial analysis of gene expression
